# A randomized community trial to advance digital epidemiological and mHealth citizen scientist compliance: A smart platform study

**DOI:** 10.1371/journal.pone.0259486

**Published:** 2021-11-01

**Authors:** Tarun Reddy Katapally, Nour Hammami, Luan Manh Chu

**Affiliations:** 1 Johnson Shoyama Graduate School of Public Policy, University of Regina, Regina, Canada; 2 Johnson Shoyama Graduate School of Public Policy, University of Saskatchewan, Saskatoon, Canada; 3 College of Medicine, Health Science Building, University of Saskatchewan, Saskatoon, Canada; 4 Institute for Health and Social Policy, McGill University, Montreal, Canada; 5 Canadian Centre for Health and Safety in Agriculture, University of Saskatchewan, Saskatoon, Canada; University of Exeter, UNITED KINGDOM

## Abstract

**Background:**

This study aims to understand how participants’ compliance and response rates to both traditional validated surveys and ecological momentary assessments (EMAs) vary across 4 cohorts who participated in the same mHealth study and received the same surveys and EMAs on their smartphones, however with cohort-specific time-triggers that differed across the 4 cohorts.

**Methods:**

As part of the Smart Platform, adult citizen scientists residing in Regina and Saskatoon, Canada, were randomly assigned to 4 cohorts in 2018. Citizen Scientists provided a complex series of subjective and objective data during 8 consecutive days using a custom-built smartphone application. All citizen scientists responded to both validated surveys and EMAs that captured physical activity. However, using Smart Platform, we varied the burden of responding to validated surveys and EMAs across cohorts by using different time-triggered push notifications. Participants in Cohort 1 (n = 10) received the full baseline 209-item validated survey on day 1 of the study; whereas participants in cohorts 2 (n = 26), 3 (n = 10), and 4 (n = 25) received the same survey in varied multiple sections over a period of 4 days. We used weighted One-way Analysis of Variance (ANOVA) tests and weighted, linear regression models to assess for differences in compliance rate across the cohort groups controlling for age, gender, and household income.

**Results:**

Compliance to EMAs that captured prospective physical activity varied across cohorts 1 to 4: 50.0% (95% Confidence Interval [C.I.] = 31.4, 68.6), 63.0% (95% C.I. = 50.7, 75.2), 37.5% (95% C.I. = 18.9, 56.1), and 61.2% (95% C.I. = 47.4, 75.0), respectively. The highest completion rate of physical activity validated surveys was observed in Cohort 4 (mean = 97.9%, 95% C.I. = 95.5, 100.0). This was also true after controlling for age, gender, and household income. The regression analyses showed that citizen scientists in Cohorts 2, 3, and 4 had significantly higher compliance with completing the physical activity validated surveys relative to citizen scientists in cohort group 1 who completed the full survey on the first day.

**Conclusions & significances:**

The findings show that maximizing the compliance rates of research participants for digital epidemiological and mHealth studies requires a balance between rigour of data collection, minimization of survey burden, and adjustment of time- and user-triggered notifications based on citizen or patient input.

## Introduction

As digital epidemiological and mobile health (mHealth) approaches are gaining momentum [1], advanced population health measurement techniques such as ecological momentary assessments (EMAs) are being increasingly used as tools to collect real-time behavioral and outcome data in real-world settings [2]. EMAs can range from subjective survey instruments to mixed-methods tools that assess population behaviors and outcomes in real-time [3,4]. In general, EMAs are deployed via mobile devices such as smartphones using time-, location, or movement-triggers (i.e., digital nudges/notifications) [3]. For example, beneficial behaviors such as a physical activity or detrimental behaviors such as addictions can be captured in real-time based on specific time of the day (i.e., 10AM/10PM) as well as location (i.e., home/workplace) and movement (i.e., walking/sitting) of the participants.

Participant compliance is critical to the success of real-time engagement with EMAs as this will inadvertently influence reliability and validity of these measures [4]. Participant compliance to EMAs can be defined as consistent and timely responses to real-time triggers throughout the duration of the study. As noncompliance behaviors can result in bias, EMA-based digital epidemiological studies should embed design and logistical protocols that improve participant compliance [3,4].

For instance, EMA study designs would require processes to minimize burden on participants, while ensuring reliable, valid, and unbiased data [4]. In order to develop evidence- based processes, it is important to understand factors that influence participant compliance. Studies which pinpoint factors that can affect compliance rates have been conducted in specific groups, including substance users, and patients with chronic pain, among others [5,6]. Current evidence indicates that participant compliance is associated with contextual [7] (location of research, social context, activity, etc.), personal [5] (age, gender, mood etc.), and logistical factors [8] (burden on participants due to EMA frequency, total length of the assessment period etc.).

However, there is a critical gap in evidence in how participant compliance can influence complex digital epidemiological studies that combine traditional validated survey measures with EMAs and objective mobile sensor data to triangulate empirical evidence [3]. With a particular focus on physical activity and sedentary behaviour, this study aims to understand how participant compliance to both traditional validated surveys and EMAs differed based on varying survey and EMA triggering mechanisms across four cohorts that participated in the same mHealth initiative [3].

## Methods

### Design

In this randomized community trial, 4 cohorts were recruited from two universities in the province of Saskatchewan, Canada—University of Saskatchewan and University of Regina. Data were obtained between January 4 and March 31, 2018, as part of the Smart Platform [3,9] a digital epidemiological and citizen science platform for ethical surveillance, integrated knowledge translation, and policy and behavioral interventions. Smart Platform’s detailed description of methods, including citizen engagement, recruitment, data collection strategies, and data privacy and security protocols have been described in the platform’s methodology publication [3]. Ethics approval was obtained from the Universities of Regina and Saskatchewan through a synchronized review protocol (REB # 2017–29).

### Participants

Participants in the Smart Platform are “citizen scientists” as they engage with the researchers at all stages of the research process. Citizen scientists informed the design, research questions and outcome measures of this study. Our citizen engagement is governed by a Citizen Scientist Advisory Council, consisting of citizens of varied age cohorts (13–18, 18–25, 25–50, >50 years), genders, ethnicities, and socioeconomic status from Saskatchewan, Canada. The Advisory Council informs conceptualization, implementation, and evaluation of Smart Platform studies. More importantly, in this study, every citizen who participated, had the ability to directly engage with the research team using user-triggered EMAs to provide their perception of data collection, integrated knowledge translation, and policy implications.

For instance, during one of our initial data collection drives, some citizens informed us that the time-triggered surveys expired too soon for them to respond in time. We were able to extend the expiration of time-triggered EMAs in near-real time. Finally, as integrated knowledge translation is part of the Smart Platform [3], results were disseminated throughout the study period using the community voices webpage of the Platform’s website [9], and time-triggered study-specific knowledge translation notifications were sent directly to citizen scientists.

### Recruitment

Adult citizen scientists who attended the Universities of Saskatchewan and Regina (i.e., undergraduate, and graduate students) were recruited via social media advertisements and recruitment lectures for this study. First, we used a social media campaign to raise awareness regarding the Smart Platform, which directed interested participants to visit our website to learn more about the study [3]. Second, after getting permission from the University administration, and instructors of randomly selected classes, research assistants from the Digital Epidemiology and Population Health Laboratory at the University of Regina, which implements the Smart Platform, visited 4 classrooms consisting of 136 students at both Universities to randomly assign citizen scientists to four cohorts. Research assistants conducted comprehensive presentations explaining the purpose, objectives, and approach of the Smart Platform. Research assistants also answered questions related to data privacy, anonymity, and responsibilities of citizen scientists.

Citizen scientists were informed that as part of the initiative they would have to use their own smartphones to download a custom-built digital epidemiological application (app), Ethica (Ethica Data Services Inc., Toronto, Canada), that was specifically adapted for the Smart Platform. The responsibilities of the citizen scientists included answering traditional validated surveys and EMAs over a period of 8 days after downloading the app, which captured data through both Android and iOS platforms. Students who agreed to contribute were guided to download Ethica.

In each class, 4 study numbers were provided to citizen scientists. They had the choice to pick one study number out of four without interacting or negotiating with other participants, which allowed them to randomly join their specific cohorts via the smartphone app. That is, after downloading the app, each citizen scientist entered their study number of choice to join their respective cohort. All citizen scientists provided informed consent through the app and confirmed their age (≥18 years) before joining the study. On the first day of the 8-day study period, all citizen scientists were provided a one-week pass to a community fitness center as an incentive for contributing to the Platform, however only <5% of the citizen scientists utilized the pass.

### Measures

All four cohorts completed the Smart survey, a 209-item integrated questionnaire that combined validated self-report surveys to record physical activity, sedentary behavior motivation, eudaimonic well-being, and perception of outdoor and indoor environment: Retrospective physical activity data were collected using the 27-item International Physical Activity Questionnaire (IPAQ), which measures activity domains [10,11]. The 9-item Sedentary Behavior Questionnaire was adapted to measure different types of screen time and non-screen time–based sedentary behavior on weekdays and weekends [12]. To capture the complexity of screen time accumulation, citizen scientists were given the option of recording common screen time behaviors (television, Internet surfing, and video games) over a range of digital devices (computer, laptop, tablet, smartphone, etc.).

Individual motivation and eudaimonic well-being were collected using the 16-item Motivation for physical activity Questionnaire [13] and the 21-item Questionnaire for Eudaimonic Well-being [14]. Perception of outdoor built environment was measured by using the 17-item Physical Activity Neighborhood Environment Survey [15]. Utilizing evidence from emerging studies [16], an indoor built environment survey was developed and used to capture participants’ perception of the indoor environment at home, work, and any fitness center associated with the participant.

**Fig 1:**
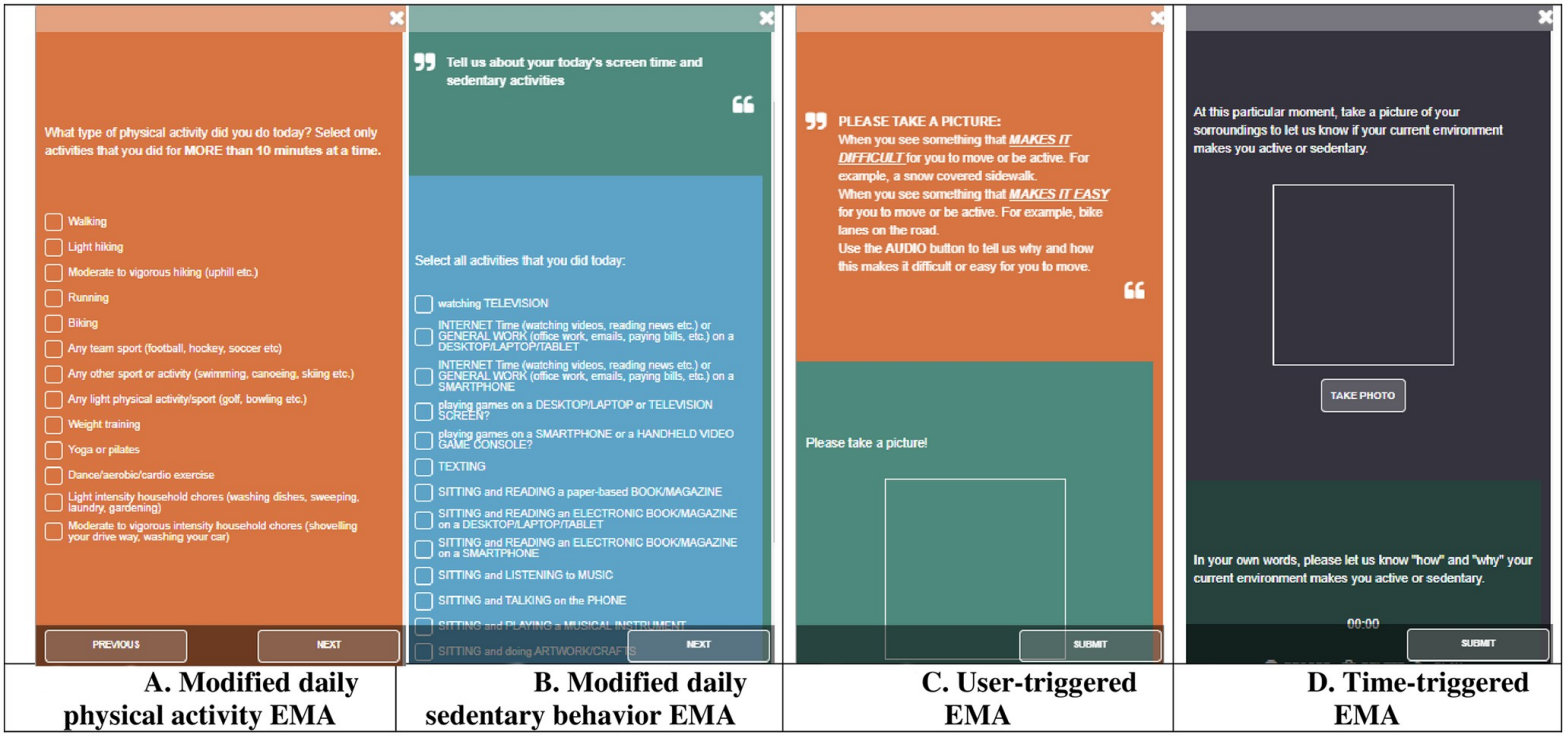
Description of deployed ecological momentary assessments (EMAs).

All four cohorts also answered time- and user- triggered EMAs on each day of the 8-day study period to capture prospective physical activity, sedentary behavior, and barriers and facilitators to physical activity using geo-coded pictures and audio files (Fig 1A–1D). Time-triggered EMAs were set to deploy at specific times during each day, whereas user-triggered EMAs were deployed by citizen scientists themselves at will. Citizen scientists had the control to trigger user-triggered EMAs at any time during the study period by opening the app and swiping on the user-triggered EMA, which was always visible to them on the main page of the app. Time-triggered modified EMAs captured prospective daily physical activity and sedentary behavior as well as social and physical contexts within which these activities occur. These modified EMAs were triggered at 8PM every day and were set to expire at midnight to allow citizen scientists to report daily behaviors. Time- and user- triggered EMAs also allowed participants to take pictures, and record audio to capture barriers and facilitators of active living across 8 days. To capture barriers and facilitators of active living, time-triggered EMAs were deployed randomly once per day, while the user-triggered EMAs were initiated by the citizen scientists anytime they desired. A comprehensive review of the measures can be found in the methodology publication of the Smart Platform [3].

### Operational definitions

Completion rates were defined as the proportion of questions that were answered divided by the number of questions in the questionnaire. Time-triggered EMA compliance rates were defined as the proportion of EMA responses divided by the number triggers. User-triggered EMA compliance was defined as the number of times citizen scientists in each cohort self-triggered EMAs and completed them.

### Data and risk management

To ensure confidentiality, data were encrypted before being stored on the smartphones and streamed to servers when devices established Wi-Fi connection. Any identifiable artefacts (e.g., photos) were removed or deidentified before data analysis. Permissions built into the Ethica app are restricted so that the app cannot access personally identifiable information that is present on the smartphones (e.g., contact list or network sites visited). MAC address anonymization was used to protect citizen scientists’ data based on a simple hash algorithm. Risks and privacy management options were made clear to citizen scientists while obtaining informed consent. All citizen scientists had the option to drop out of the study or pause data gathering anytime they wished via the app. Moreover, they also had the option in the settings of the app to upload data only when they had WI-FI access and/or when they were charging their phones [3]. Clear instructions were provided regarding study withdrawal within the app.

### Statistical analyses

Sample size and power calculations for the study design showed that a minimum of 12 individuals (3 per arm) would be needed to test the null hypothesis that the population mean is the same across all groups. The power of this suggested sample size was estimated at 0.852 based on a noncentral F-distribution and a significance level of 5%. As for the power of our study (n = 88), it was estimated at >0.999 based on a noncentral F-distribution and a significance level of 5%.

Additionally, for all analyses, we used analytical weights across all four cohorts; as such, each cohort (regardless of the sample size) contributed to 25% to a total of 100% to the analyses.

Continuous estimates were reported as weighted means with standard deviations (SD). Categorical estimates were reported as weighted percentages. Weighted prevalence of the completion rates for the time-triggered EMAs and the user-triggered EMAs were reported across cohort groups. We also assessed the weighted completion rate for the physical activity EMA and the IPAQ across cohort groups. For this analysis we conducted spearman correlation procedures to assess the correlation between the compliance rates between traditional validated survey measure for physical activity (IPAQ survey) and adapted EMA measurements and how they differed across cohort groups. One-way Analysis of Variance (ANOVA) tests were used to identify differences across cohort groups.

Additionally, to further understand differences in the IPAQ survey and EMA completion rates across cohort groups, we conducted weighted, linear regression models that controlled for age, gender, and household income. Findings from these models provide evidence as to the association between cohort group design and the citizen scientists’ respective completion rate, that cannot be attributed to citizen scientists’ age, gender, or household income as these were controlled for in the models. Analyses were conducted in Stata version 15.0 [17] with significance set at an alpha of < 0.05.

## Results

In total, out of 136 students in the four classes that we approached for recruitment, 88 students agreed to participate as citizen scientists in this trial. Citizen scientists completed IPAQ as well as EMAs, which ranged from modified daily evening EMAs to capture physical activity and sedentary behavior to user-and time-triggered geo-coded EMAs that allowed citizen scientists to report barriers and facilitators of physical activity using their smartphone camera and audio functions (Fig 1). Out of these 88 participants, 71 provided valid information on age, gender, and education. Table 1 shows both weighted and unweighted demographic characteristics of citizen scientists across all cohorts. The mean age of the total study sample was 33.1 years (SD = 15.1), out of which 73.7% were females (n = 52).

**Table 1:** Weighted summary statistics for the sample and per cohort group.

	Cohort 1	Cohort 2	Cohort 3	Cohort 4	Total
**Unweighted sample**	14.1 (n = 10)	36.6 (n = 26)	14.1 (n = 10)	35.2 (n = 25)	100 (n = 71)
**Weighted sample**	25 (n = 17.7)	25 (n = 17.7)	25 (n = 17.7)	25 (n = 17.7)	100 (n = 71)
	Weighted mean and standard deviation				
**Age in years**	43.9 (16.7)	24.8 (8.3)	31.6 (15.1)	32.2 (14.0)	33.1 (15.1)
	Weighted percent				
**Gender (n = 71)**					
Males (n = 19)	20	23.1	30	32	26.3
Females (n = 52)	80	76.9	70	68	73.7
**Education (n = 71)**					
Some secondary/high school (n = 1)	0	0	10	0	2.5
Completed secondary/high school (n = 6)	20	7.7	10	4	10.4
Some post-secondary (university or college) (n = 35)	0	69.2	40	52	40.3
Received university or college degree/diploma (n = 29)	80	23.1	40	44	46.8
**Household income (n = 69)**					
< 40,000 (n = 20)	10	50	0	24	22.1
40,000- <100,000 (n = 19)	30	15.4	50	32	30.9
>or = 100,00 (n = 30)	60	34.6	50	44	47

**Fig 2:**
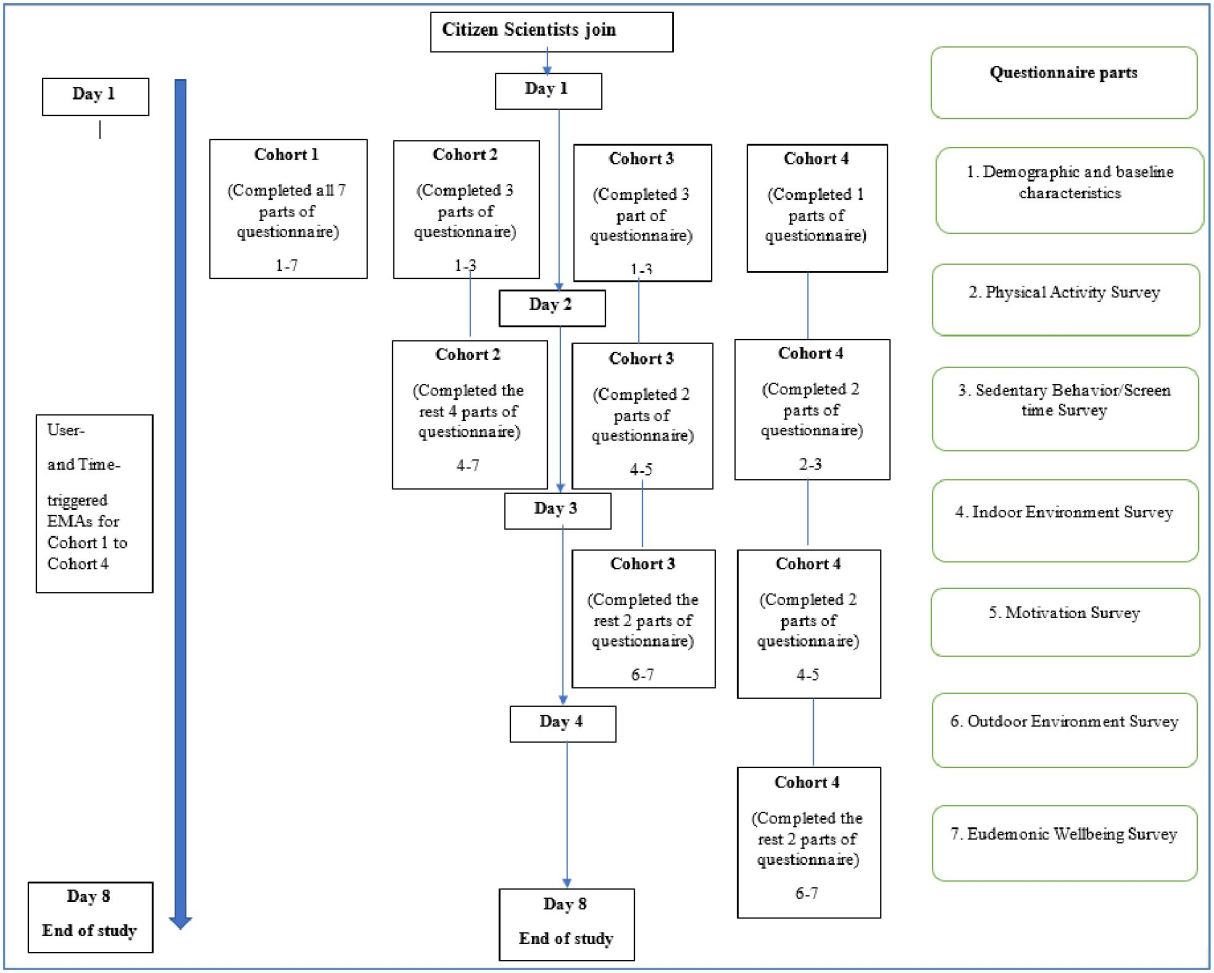
Smart survey and ecological momentary assessment deployment timeline across all cohorts.

Fig 2 shows differences in the deployment of the Smart survey across the cohorts. Citizen scientists in cohort 1 completed the 209-item Smart survey on day 1 of the study. This consisted of seven parts: (1) demographic and baseline characteristics, (2) physical activity survey (IPAQ), (3) sedentary behavior/screen time survey, (4) indoor environment survey, (5) motivation survey, (6) outdoor environment survey, and (7) eudemonic wellbeing survey. Citizen scientists in cohort 2 received parts 1, 2, and 3 on the first day and parts 4, 5, 6, and 7 on the second day. Citizen scientists in cohort 3 received parts 1, 2, and 3 on the first day, parts 4, and 5, on the second day, and parts 6 and 7 on the third day. Citizen scientists in cohort 4 received part 1 only on the first day, parts 2 and 3 on the second day, parts 4 and 5, on the third day, and parts 6 and 7 on the fourth day. User- and time-triggered EMA deployment and triggers were identical across all cohorts.

Table 2 displays the weighted prevalence of citizen science compliance with EMAs capturing active living barriers and facilitators—time-triggered and user-triggered EMAs per cohort group. Compliance rates were calculated as the proportion of EMA responses divided by the number of triggers. ANOVA tests detected that although citizen scientists had higher compliance with user-triggered EMAs (67.1%) than time-triggered EMAs (32.9%), no significant differences in compliance rates were observed across cohorts.

**Table 2:** Weighted prevalence of citizen scientist compliance with EMAs capturing active living barriers and facilitators: Time-triggered EMAs ^a^ and user- triggered EMAs ^a^ per cohort group in percent.

	Time-triggered EMA ^a^	User-triggered EMA ^a^
	% (95% CI)	% (95% CI)
Overall	32.9 (26.6, 39.2)	67.1 (62.7, 71.5)
Cohort 1	30.3 (18.3, 42.4)	79.2 (74.7, 83.6)
Cohort 2	32.7 (22.0, 43.4)	62.8 (56.6, 68.9)
Cohort 3	35.7 (12.9, 58.5)	54.8 (40.7, 70.9)
Cohort 4	32.7 (21.9, 43.6)	71.5 (66.6, 76.6)

^a^ EMAs: Ecological momentary assessments.

Table 3 displays the weighted prevalence of citizen science compliance with the IPAQ and daily physical activity EMAs, and their corresponding correlation coefficients per cohort group. Across all cohorts, the IPAQ had a high compliance rate (89.8%) while daily physical activity EMA compliance rates were lower at 52.9%. As for differences across the cohorts, citizen scientists in cohort 4 had the highest IPAQ compliance rate (97.9%).

**Table 3:** Weighted prevalence of citizen scientist compliance with IPAQ ^a^ and with daily PA EMA ^b^ and their corresponding correlation coefficients per cohort group in percent.

	IPAQ ^a^	Daily PA EMA ^b^	Correlation coefficients
	% (95% CI)	% (95% CI)	
Overall	89.8 (86.2, 93.5)	52.9 (45.3, 60.6)	0.40**
Cohort 1	79.9 (76.9, 83.0)	50.0 (31.4, 68.6)	-0.58
Cohort 2	95.3 (89.7, 100.0)	63.0 (50.7, 75.2)	0.50*
Cohort 3	86.2 (72.6, 99.9)	37.5 (18.9, 56.1)	0.47
Cohort 4	97.9 (95.5, 100.0)	61.2 (47.4, 75.0)	0.58**

^a^ IPAQ: International physical activity questionnaire.

^b^ EMA: Ecological momentary assessment.

*** p<0.0001,

** p<0.01,

*p<0.05.

**Fig 3:**
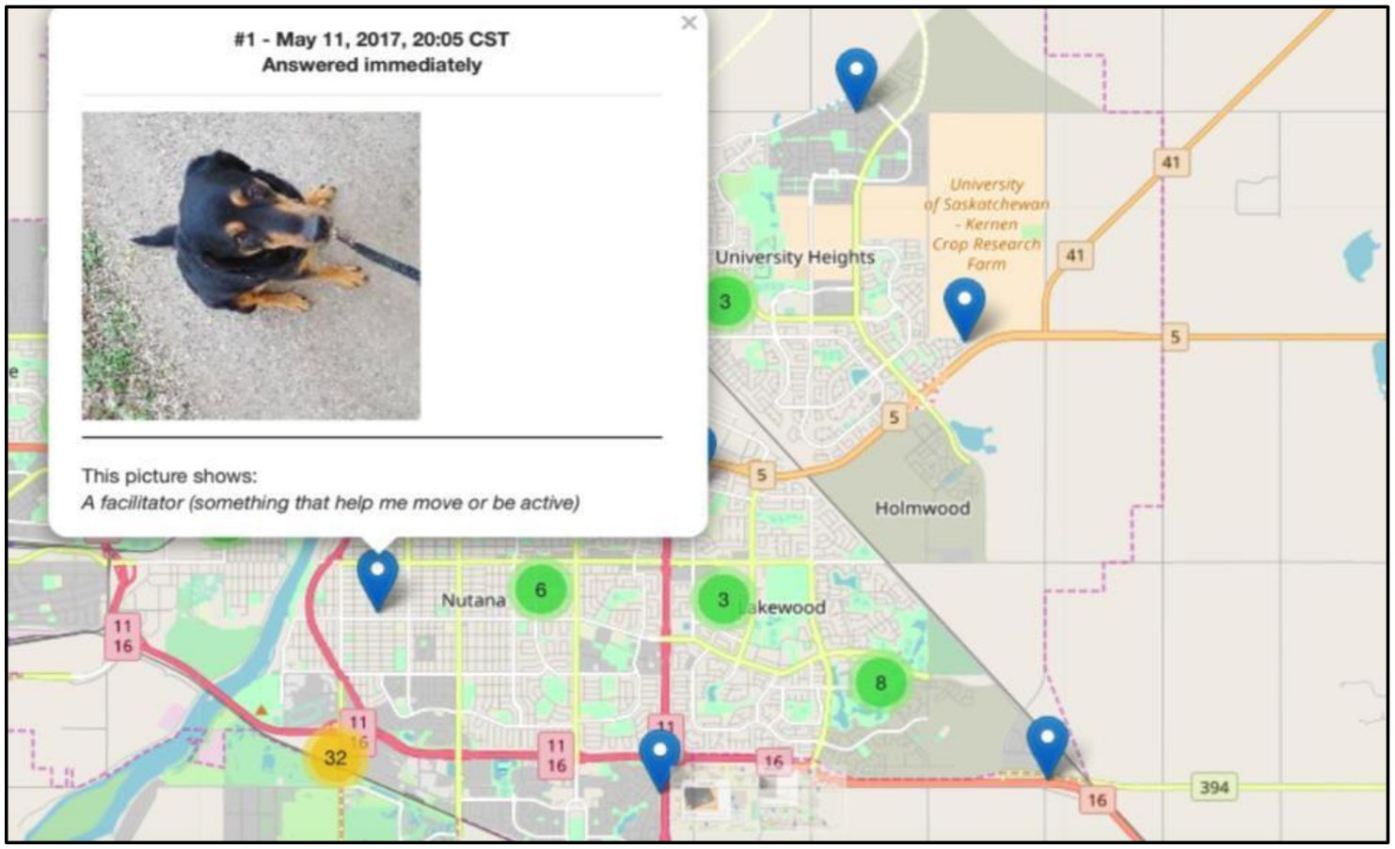
Geocoded user- and time-triggered ecological momentary assessments capturing citizen scientist perceptions in real-time.

Citizen scientists in cohort 2 had the highest daily physical activity EMA compliance rate (63.0%). Citizen scientists in cohort 1 had the lowest IPAQ compliance rate (79.9%) and a low daily physical activity EMA compliance rate (50.0%), relative to citizen scientists in the other cohorts. The IPAQ and daily physical activity EMAs were significantly correlated among the overall sample; however, cohort-specific correlations show that IPAQ and daily physical activity EMA compliance was significantly correlated only among cohorts 2 and 4. Apart from EMAs that captured physical activity and sedentary behaviour, citizen scientists were also able to report their perception of physical activity barriers and facilitators in real-time using geocoded user- and time-triggered EMAs (Fig 3).

Table 4 displays results from the weighted, linear regression models assessing the association between cohorts and their compliance rates for time-triggered active living barrier and facilitator capturing EMAs (Model 1), user-triggered active living barrier and facilitator capturing EMAs (Model 2), the IPAQ (Model 3), physical activity EMAs (Model 4), and sedentary behavior EMAs (Model 5), while controlling for age, gender, and household income. Results from model 1 show that there are no significant differences in compliance with time-triggered active living barrier and facilitator capturing EMAs across cohorts. Results from model 2 show that, compared with cohort 1, cohorts 2 and 3 have lower compliance with user triggered active living barrier and facilitator capturing EMAs. Results from model 3 show that cohorts 2, 3, and 4 have significantly higher compliance with IPAQ relative to cohort 1. Results from model 4 show that there are no significant differences across cohorts in compliance with physical activity EMAs, however, results from model 5 show that cohorts 2 and 3 have lower compliance with sedentary behavior EMAs in comparison with cohort 1.

**Table 4:** Weighted beta coefficients (95% Confidence intervals) estimating the association between citizen scientist’s cohort groups and their compliance with: Time-triggered EMA ^a^, user-triggered EMA ^a^, IPAQ ^b^, PA EMA ^a^, and sedentary behaviour EMA ^a^ while controlling for age, gender, and household income.

	Model 1	Model 2	Model 3	Model 4	Model 5
	Time-triggered EMA ^a^	User-triggered EMA ^a^	IPAQ ^b^	Physical activity EMA ^a^	Sedentary behavior EMA ^a^
Cohort 1 (Ref.)					
Cohort 2	0.11 (-0.12, 0.35)	-0.15 * (-0.28, -0.023)	0.17*** (0.089, 0.25)	0.13 (-0.12, 0.39)	-0.16 ** (-0.27, -0.043)
Cohort 3	0.16 (-0.079, 0.39)	-0.13 * (-0.25, -0.0054)	0.13** (0.046, 0.21)	-0.065 (-0.31, 0.18)	-0.13 * (-0.24, -0.012)
Cohort 4	0.077 (-0.14, 0.29)	-0.084 (-0.20, 0.036)	0.20 *** (0.12, 0.28)	0.12 (-0.12, 0.36)	-0.081 (-0.18, 0.026)

All models controlled for age, gender, and household income.

^a^ EMA: Ecological momentary assessment.

^b^ IPAQ: International physical activity questionnaire.

*** p<0.0001,

** p<0.01,

*p<0.05.

## Discussion

This study aims to understand how completion rates of traditional validated surveys, and compliance rates of EMAs vary across four cohorts of citizen scientists who participated in the same mHealth initiative [3] and received the same validated surveys and EMAs on their smartphones. However, the key difference between the 4 cohorts was the deployment of the 209 item Smart survey, which was segmented using time-triggered notifications. The rationale behind this approach was twofold: 1) To understand how citizen scientists’ compliance varies when comprehensive digital epidemiological and population health studies are conducted by deploying traditional validated survey measures with EMAs and objective mobile sensor data collection to triangulate empirical evidence; 2) To empirically determine if utilizing different time triggers to deploy burdensome traditional validated surveys [18] would change completion rates of not only the validated surveys, but also compliance rates of user- and time-triggered EMAs.

Evidence indicates that most EMA studies have been conducted in disease-specific populations, where compliance rates ranged between approximately 70% to 85% [8]. Our findings indicate similar rates for the completion rates of traditional validated surveys, when deployed based on various time-triggers. Our study adds that completion rates increased with reduction of burden i.e., shorter segments of validated surveys deployed with pre-defined time time-triggers, irrespective of the type and level of segmentation improved completion rates in comparison with non-segmented measures. For instance, all cohorts in which time-triggered 209-item Smart survey was deployed to segment it across several days showed greater completion of validated measures in comparison with Cohort 1, where the 209-item Smart survey was deployed at the same time on day 1 of the study. This is novel and interesting finding that shows that deploying traditional validated surveys with a time-triggered EMA approach can increase compliance even in complex and burdensome digital epidemiological studies.

However, there is a fine line. Our regression analyses showed that cohorts 2 and 3 had lower compliance with user triggered EMAs and time-triggered daily sedentary behaviour EMAs relative to cohort 1. A possible explanation is, since citizen scientists in cohorts 2 and 3 completed the 209-item Smart survey over several days, they might have neglected EMAs, which require additional motivation for completion. This suggests that EMAs when deployed with traditional validated measures require careful balancing of participant burden.

This may also explain why our modified EMAs that captured daily physical activity and sedentary behavior showed lower compliance rates (50% to 63%) in comparison with other studies [19]. For instance, a recent systematic review reported the average compliance rate of EMAs was 77%, with individual study rates ranging from 56 to 97.7% [19]. The length of our EMAs were in line with these studies taking between less than a minute to a maximum of 5 minutes for completion [8,19].

EMA studies capture varied behaviors ranging from sleep and physical activity to addictions and pain, with various degrees of burden in terms of longevity and frequency of EMAs. The modified EMAs that captured daily physical activity and sedentary behavior were potentially more burdensome as they required citizen scientists to answer a short but complex survey that required recall of not only types of physical activities and sedentary behaviors, but also the physical (e.g., home, parks) and social contexts (e.g., with friends, family) within which these behaviors occurred.

Moreover, the population under investigation could be a major determinant of compliance as well. For instance, the sole motivation of our sample, which was recruited from a University population (i.e., apparently asymptomatic population), was to provide data for the greater purpose of understanding population active living behaviors, while symptomatic individuals (i.e., patients) are potentially more likely to adhere to the EMA study protocols because they understand the immediate benefits of participation, which include improved health outcomes [16,20]. It is important to understand unique conditions of each EMA study protocol to set expectations and maximize compliance.

There are several logistical and design approaches that could enhance compliance of EMA- based studies, which range from providing adequate participant incentives to testing and piloting for optimum balance between EMA deployment time, trigger frequency, and overall study period [15,16]. However, till date, no studies have been conducted to comprehensively test complexities of EMA compliance by taking into consideration not only different types of EMAs and varied triggering processes, but also the temporal EMA deployment combined with variations of deployment of traditional validated surveys. For instance, in our consecutive 8-day study we deployed two different types of EMAs—modified time-triggered EMAs that captured daily physical activity and sedentary behavior, and time- and user-triggered EMAs that captured citizen scientist perceptions of geo-coded barriers and facilitators of physical activity and sedentary.

These two types of EMAs not only differed in their types, but also complexity, with daily physical activity and sedentary behavior EMAs focusing on the quantity and context of physical activity, where the geo-coded EMAs capturing barriers and facilitators of physical activity by leveraging camera and audio functions of citizen-owned smartphones. More importantly, our study is the first to deploy validated surveys in varying lengths and time-triggers across four different cohorts to understand participant compliance in complex digital epidemiological and mHealth studies that can utilize a combination of validated surveys and sensor-based EMAs to triangulate population health behaviors and outcomes.

The evidence we generated by this rigorous approach has one consistent pattern—when burdensome traditional validated surveys are deployed with different types of EMAs, the completion rate of validated surveys increased among cohorts in which validated surveys were segmented and time-triggered over a period of 4 days. However, EMAs have higher compliance when participants do not have lengthy validated survey measures to complete. This pattern was true for both time-triggered EMAs that captured daily sedentary behavior, and user-triggered EMAs that captured citizen scientists’ perception of physical activity barriers and facilitators. Although there is some evidence that long duration of EMA studies is associated with lower completion rates [21], the evidence is not conclusive yet [5].

Ono et al. [21] conducted a meta-analysis to investigate factors affecting EMA-completion rates among 701 patients with chronic pain from 10 studies and found that on average, completion rates decreased by approximately 2.0% per week of EMA sampling. However, in a systematic review conducted by Wen et al., [22] to investigate youth compliance to mobile-EMA protocols, evidence showed that compliance rates did not differ by study duration among both clinical or nonclinical settings.

Nevertheless, the evidence from our study indicates that an important factor associated with EMA compliance is participant burden, which can be minimized by effective triggering of EMAs. The findings our study indicate that an appropriate study protocol should balance the need between using different types of EMAs (time-triggered and user-triggered) and traditional validated measures to address the necessary research questions. An ideal scenario would be to validate EMA measures so that burdensome traditional validated measures could be avoided altogether in the future [4].

### Strengths and limitations

The primary strength of our study is the study design, which enabled citizen scientists to randomly assign themselves to different cohorts to minimize bias. Moreover, as we used a citizen science approach to leverage citizen-owned devices working on both iOS and Android platforms, the study has extensive generalizability and external validity. Finally, the complex deployment of both validated surveys and different types of EMAs temporally and consecutively across 8 days provides this study methodological rigour to understand complexities of participant compliance. One limitation of this study is the small sample size and lack of data on why citizen scientists did not complete or comply with validated surveys and EMAs. We also focused only on IPAQ in terms of a validated physical activity measure because all EMAs were specific to either physical activity or sedentary behaviour measurement. Future studies should try to replicate our approach with larger sample sizes including other validated measures and potentially obtain both quantitative and qualitative data to ascertain the cause of low compliance and completion rates.

## Conclusions

This is the first experimental digital epidemiological and mHealth study that used a citizen science approach to deploy both traditional validated surveys and different types of time- and user- triggered EMAs through citizen-owned smartphones to understand completion rates of validated surveys and compliance rates of EMAs. While the evidence indicates there wasn’t a dose-response relationship between reduction of burden and compliance across 4 cohorts; our preliminary findings show that, the key determinant of completion and compliance rates for traditional validated surveys in digital epidemiological and mHealth studies is intuitive time-triggers that segment the surveys and reduce burden. However, when combining traditional surveys with EMAs, it is important to balance compliance of both types of measures to address the relevant research questions. The approach employed in this study should be replicated across with larger sample sizes using citizen science techniques to confirm these findings and pave the way for digital epidemiological and mHealth studies to ethically leverage big data.
